# Investigating the Interactions of Glioma Stem Cells in the Perivascular Niche at Single‐Cell Resolution using a Microfluidic Tumor Microenvironment Model

**DOI:** 10.1002/advs.202201436

**Published:** 2022-05-26

**Authors:** Emmanuela A. Adjei‐Sowah, Samantha A. O'Connor, Jaimeson Veldhuizen, Costanza Lo Cascio, Christopher Plaisier, Shwetal Mehta, Mehdi Nikkhah

**Affiliations:** ^1^ School of Biological and Health Systems Engineering Arizona State University Tempe AZ 85287‐9709 USA; ^2^ Ivy Brain Tumor Center, Barrow Neurological Institute St. Joseph's Hospital and Medical Center 350 W Thomas Rd Phoenix AZ 85013 USA; ^3^ Virginia G. Piper Biodesign Center for Personalized Diagnostics Arizona State University Tempe AZ 85287‐9709 USA

**Keywords:** glioblastoma, invasion, perivascular niche, transcriptomics, tumor microenvironment

## Abstract

The perivascular niche (PVN) is a glioblastoma tumor microenvironment (TME) that serves as a safe haven for glioma stem cells (GSCs), and acts as a reservoir that inevitably leads to tumor recurrence. Understanding cellular interactions in the PVN that drive GSC treatment resistance and stemness is crucial to develop lasting therapies for glioblastoma. The limitations of in vivo models and in vitro assays have led to critical knowledge gaps regarding the influence of various cell types in the PVN on GSCs behavior. This study developed an organotypic triculture microfluidic model as a means to recapitulate the PVN and study its impact on GSCs. This triculture platform, comprised of endothelial cells (ECs), astrocytes, and GSCs, is used to investigate GSC invasion, proliferation and stemness. Both ECs and astrocytes significantly increased invasiveness of GSCs. This study futher identified 15 ligand‐receptor pairs using single‐cell RNAseq with putative chemotactic mechanisms of GSCs, where the receptor is up‐regulated in GSCs and the diffusible ligand is expressed in either astrocytes or ECs. Notably, the ligand–receptor pair SAA1‐FPR1 is demonstrated to be involved in chemotactic invasion of GSCs toward PVN. The novel triculture platform presented herein can be used for therapeutic development and discovery of molecular mechanisms driving GSC biology.

## Graphical abstract

An organotypic triculture microfluidic model is developed to recapitulate the perivascular niche in glioblastoma (GBM) and study its impact on glioma stem cells (GSCs) behavior. Endothelial cells and astrocytes synergistically influence invasiveness of GSCs. Single‐cell RNA‐seq identifies the ligand‐receptor pair, SAA1‐FPR1, which is involved in chemotactic invasion of GSCs toward the perivascular niche.

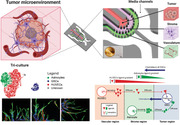

## 1 Introduction

Glioblastoma multiforme (GBM) is the most common, aggressive, and invasive primary brain cancer in adults.^[^ ^1^, ^2^ ^]^ The median patient survival remains 14–16 months despite surgical resection combined with radiation plus chemotherapy, and the tumors almost always recur.^[^ ^3^ ^]^ Treatment failures have been attributed to several factors including extensive inter‐ and intratumoral heterogeneity and complex interactions within the tumor microenvironment (TME) that drive therapy resistance.^[^ ^4^, ^5^, ^6^, ^7^, ^8^ ^]^ A subpopulation of tumors cells with stem‐like properties, glioma stem cells (GSCs), have been shown to be inherently radio‐ and chemoresistant^[^ ^3^, ^9^, ^10^ ^]^ and is one of the reasons for tumor recurrence and therapy resistance. Similar to neural stem cells, GSCs can self‐renew and differentiate into different cell types of the neuro‐glial lineage^[^ ^11^ ^]^ and reside within specialized microenvironments that serve as protective niches.^[^ ^11^ ^]^ The perivascular niche (PVN) is one of the most critically studied sites and has been shown to promote stemness, invasion, and therapy resistance of GSCs.^[^ ^12^, ^13^ ^]^ Previous studies using in vitro and in vivo models have demonstrated that the crosstalk between the endothelial cells (ECs) and GSCs regulates GSC proliferation,^[^ ^2^, ^14^ ^]^ tumorigenicity,^[^ ^15^, ^16^, ^17^, ^18^ ^]^ and self‐renewal capacity.^[^ ^19^, ^20^ ^]^ However, the PVN is a complex microenvironment comprised not only of ECs but multiple other cell types including astrocytes, pericytes, immune, and other stromal cells that regulate GSC biology.^[^ ^2^, ^12^ ^]^ How various cellular components of the PVN alter GSC behavior (proliferation versus quiescence and invasion versus homing) and affect therapy resistance is not well understood. This is in part due to the limitations of conventional 2D and 3D in vitro systems as well as in vivo murine xenograft models. These model systems are not well suited to control critical aspects of the PVN that are necessary to dissect the effect of individual niche‐specific cell types on GSC behavior.

The critical role of the PVN in the promotion of tumor progression has motivated efforts to develop advanced biomimetic in vitro models to recapitulate the complexities of this critical niche (PVN) within the GBM TME. Tissue‐engineered and microfluidic models have gained significant attention in this regard.^[^ ^15^, ^16^, ^17^, ^21^, ^22^, ^23^, ^24^ ^]^ Tissue‐engineered approaches have utilized brain extracellular matrix (ECM) components, mainly hyaluronic acid mixed in gelatin^[^ ^21^, ^25^, ^26^, ^27^, ^28^ ^]^ and collagen,^[^ ^29^, ^30^ ^]^ to construct 3D cell‐laden hydrogel constructs to mimic the GBM TME.^[^ ^31^ ^]^ However, these model systems lack organotypic architecture and the spatial organization of various cell types to construct well‐defined TME niches. Alternatively, parallel‐channel microfluidic models integrated with hydrogel biomaterials^[^ ^16^, ^17^, ^18^, ^23^, ^24^ ^]^ have been utilized to generate physiologically relevant vascular networks to construct the PVN and assess GSC phenotype, homing, and the signaling crosstalk within the niche. A few studies have also utilized 3D bioprinting to develop the GBM PVN,^[^ ^32^, ^33^, ^34^ ^]^ in which the formation of hypoxia‐induced necrotic cores and pseudopalisades aided the assessment of tumor resistance to standard therapies in the model.^[^ ^32^ ^]^ Despite significant and important findings, the majority of these studies relied only upon coculturing of GSCs with ECs. These models did not include other supporting niche‐specific cell types, such as astrocytes, to construct a realistic and complex microenvironment model to dissect the role of other cell types in the PVN on biological functions of GSCs.

Here, we report development of a 3D organotypic microfluidic platform as an attempt to mimic the complex multicellular PVN to dissect the molecular interactions between the GSCs, stromal cells, and vascular cells, which lead to GSC invasion. Using the proposed tumor‐on‐chip platform, we formed a spontaneously assembled microvascular region and incorporated patient‐derived GSCs and astrocytes within demarcated tumor and stroma regions of the tumor model, to better mimic the biological complexities of the GBM PVN. We primarily studied the effect of triculture (GSC‐Astrocyte‐EC) interactions on GSC invasion, proliferation, and stem phenotype as compared to coculture and monoculture conditions. We found that the incorporation of astrocytes and ECs significantly increased GSC invasion. Furthermore, we performed mechanistic biological studies, utilizing single‐cell RNA sequencing (scRNA‐seq), to identify putative ligand‐receptor pairs that drive GSC invasion towards the vascular network. Notably, we discovered significant upregulation of novel receptors (i.e., LGR6 and FPR1) on the GSCs and their corresponding ligands only in the triculture condition, which have not been previously demonstrated to function in the context of GBM. We further confirmed the role of these ligand–receptor pairs on the migratory processes of GSCs. Altogether, our study presents a novel approach utilizing a physiologically relevant and an organotypic triculture GBM tumor on‐a‐chip model, integrated with scRNA‐seq, to assess the phenotype of patient‐derived GSCs within the PVN and identify novel ligand‐receptor pairs which drive GSCs invasion in presence of ECs and astrocytes.

## 2 Results

### 2.1 Formation of the Microfluidic On‐Chip Tumor Model and Vasculogenesis

**Figure 1:**
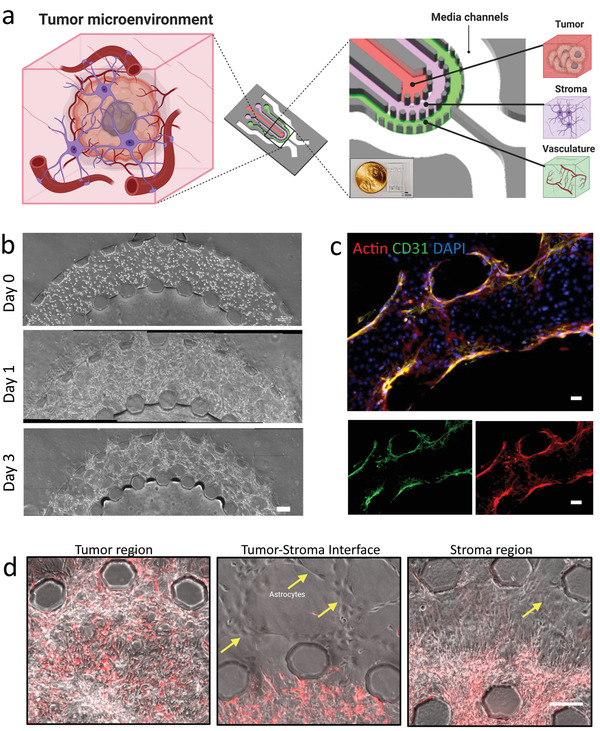
Schematic of GBM PVN developed within a microfluidic chip model. a) Schematic of PVN within a GBM TME. Components of the GBM TME are embedded into their respective regions on the microfluidic device (red: tumor region, purple: stroma region, green: vascular region). b) Phase‐contrast images of vasculature formation between day 0 and day 3. Scale bar = 100 µm. c) Fluorescent images showing formed vascular network expressing Actin and CD31 within microfluidic device. Scale bar = 20 µm. d) Phase‐contrast images showing tumor and stroma regions of microfluidic device. Yellow arrows point to astrocytes within stroma region. Scale bar: 100 µm.

The microfluidic GBM tumor‐on‐chip model consisted of three concentric cell culture regions, namely the vasculature, stroma, and tumor regions (**Figure** **1**a), surrounded by media channels. Hexagonal microposts that bound the regions were evenly spaced at 100 µm to enable the distinction of tumor, stroma, and vascular entities in an organotypic manner while keeping these regions interconnected. This enabled cellular interactions and invasion of GSCs from the tumor region to adjacent stromal region. The hexagonal shape of the microposts enabled optimal shear stress during injection to contain the hydrogel within the vascular and tumor regions without leaking into other regions.^[^ ^35^ ^]^ The channels in the device were bound by transparent coverslips which permitted real‐time 3D imaging.

Prior to construction of the tumor model and injection of all regions, we first formed a 3D microvascular network by encapsulating ECs in a fibrin hydrogel matrix in the outer region of the device (i.e., the vascular region). We monitored the formation of the vascular layer over the course of 72 h through phase‐contrast imaging every 24 h (Figure 1b) and IF staining of CD31 and phalloidin after 72 h (Figure 1c). The ECs were round within the fibrin hydrogel matrix and upon injection into the vascular region of the device (Figure 1b,c), but over time, formation of interconnected vessels was observed (Figure S1, Supporting Information). The diameters of the vessels were measured to be 48 ± 9 µm, consistent with our previous studies.^[^ ^15^ ^]^

### 2.2 Astrocytes and ECs Differentially Influence GSC Invasion within the Microfluidic Tumor Model

**Figure 2:**
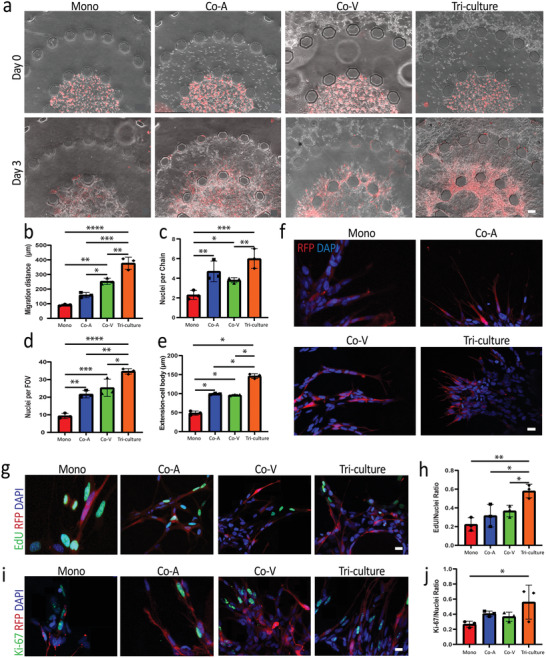
GB3‐RFP cells significantly invade into the stroma region of microfluidic device in presence of astrocytes and ECs. a) Phase‐contrast images of GB3‐RFP (red) invading stroma region in the presence of ECs and astrocytes. b) Quantification of invasion distance for each condition; Scale bar: 100 µm. c) Quantification of nuclei per chain in each experimental condition. d) Quantification of nuclei per field of view in each experimental condition. e) Quantification of GB3‐RFP cell extension in each experimental condition. f) Immunofluorescence staining of GB3‐RFP cells (red) exhibiting elongated and thin morphology when astrocytes are present in stroma layer during invasion. Scale bar: 20 µm. g) Immunofluorescence staining of EdU and h) quantification of EdU/nuclei ratio of each experimental condition. i) Immunofluorescence staining of Ki‐67 proliferative markers and j) quantification of Ki‐67/nuclei ratio of each experimental condition; Scale bar: 20 µm. (* = < 0.05; ** < 0.007; *** < 0.0004; **** < 0.0001; 2‐way ANOVA with Tukey's multiple comparison test; *n* = 3 for each data set). (Monoculture; Mono, Coculture with astrocytes; Co‐A, Coculture with vasculature; Co‐V)

To form the tumor model, we used a patient‐derived GSC line, GB3‐RFP, which was transduced with a lentivirus expressing RFP to compare the invasive capacity of the GSCs across various conditions in our tumor‐on‐chip model. After formation of the vascular layer, we injected GB3‐RFP cells into the tumor region, and astrocytes into the stromal region of the device. We sought to utilize our novel triculture model to investigate the influence of astrocytes and ECs, as well as their combinatorial effect, on GB3‐RFP biological proliferation and invasion. We established four different biological conditions: i) GB3‐RFP monoculture condition; ii) coculture with vasculature; iii) coculture with astrocytes; and iv) triculture of GB3‐RFP cells, ECs, and astrocytes (Table S1, Supporting Information). The invasion of GB3‐RFP cells across the tumor–stroma interface was observed by phase‐contrast imaging intersected with fluorescence. Within 24 h of embedding GB3‐RFP cells in the tumor region (after vasculature formation), they exhibited directed migration toward the stroma and vascular regions in all conditions except the monoculture condition, where no directional preference was observed. Images of GB3‐RFP cells in the triculture condition at 72 h revealed significant migration of these cells from the tumor region to the periphery of the vascular region (**Figure** **2**a). We also observed after 72 h that the GB3‐RFP cells in triculture condition densely populated the stroma region with marked cellular protrusions during invasion, relative to the other three conditions (i.e., monoculture, coculture with astrocytes, coculture with vasculature). Astrocytes in the stromal layer also started to spread within 24 h of insertion. By 72 h, the astrocytes had proliferated and created “migratory tracts” within the stromal region. Invasion of GB3‐RFP cells at 72 h was quantitatively compared across the four experimental conditions by determining migration distance using NIH ImageJ software. Particularly, the highest migration of GB3‐RFP cells was observed in the triculture condition (376.8 ± 55µm), followed by the coculture with vasculature condition (234.67 ± 25µm) (Figure 2b). Taken together this clearly delineates the contributions of stromal cells (i.e., astrocytes) along with vasculature in generating an aggressive phenotype in GB3‐RFP cells within the PVN, consistent with previous studies.^[^ ^36^, ^37^ ^]^

To further characterize the invasive behavior of the GB3‐RFP cells within our tumor model, we analyzed three different metrics, namely, Nuclei per Chain, Nuclei per Field of View (FOV), and Extension from Cell Body. We observed significant differences in migrating cell density when we quantified based on nuclei per chain (Figure 2c). Specifically, GB3‐RFP cells densely populated the stromal region of the tumor model when cocultured with astrocytes, and exhibited even higher density in triculture. Correspondingly, GB3‐RFP cells in the triculture and coculture with astrocytes exhibited elongated cell protrusions compared to the other conditions. Moreover, analysis of other metrics, including extension from cell body and nuclei per FOV, revealed significant differences between experimental groups, with GB3‐RFP cells in the triculture group exhibiting the longest extensions with highest cellular presence (i.e., nuclei per FOV) in the stroma region. (Figure 2d,e). These results indicate that the migratory pattern of GB3‐RFP cells was influenced by stroma and vascular network presence.

GSCs have been shown to invade surrounding tissues in specific patterns and exhibit phenotypes relative to their ECM counterparts in vivo, with longer protrusions or extensions on GSCs correlating to increased invasiveness.^[^ ^38^, ^39^, ^40^ ^]^ Therefore, we further analyzed the morphology of the GB3‐RFP cells grown in monoculture, coculture with astrocytes, coculture with vasculature, as well as triculture condition. Within 24 h after GB3‐RFP injection into the tumor region, we observed elongated and branching morphology of the GB3‐RFP cells. This morphology was present in all conditions (Figure 2f). By 72 h, GB3‐RFP cells within the triculture and coculture with astrocytes conditions appeared rather narrow and elongated, while the cells in the coculture with vasculature and monoculture conditions exhibited comparatively shorter and broader GB3‐RFP cell extensions (Figure 2f). Overall, we observed fewer, but thicker GB3‐RFP cell extensions per cell body in the monoculture and coculture with vasculature conditions, while the GB3‐RFP cells grown in triculture and in coculture with astrocytes exhibited many, but thinner cell extensions per cell body.

Previous studies have described stochastic “go or grow” patterns in highly infiltrative GBM, with proliferation and invasion being characterized as mutually exclusive.^[^ ^41^, ^42^ ^]^ Due to the increased invasion of GB3‐RFP cells in triculture condition, we further interrogated GB3‐RFP cell proliferation using both EdU and Ki‐67 staining kits and analyzed the overlap of positive expression with RFP within the stromal region of the platform. We sought to understand whether the different cellular compositions within individual experimental conditions influenced the proliferative phenotype of the GB3‐RFP cells. We observed a significantly higher number of proliferating GB3‐RFP cells, counted within the stromal region, in the tri‐culture condition, compared to the other conditions (Figure 2g,h). Similar GB3‐RFP proliferative patterns were observed when we utilized Ki‐67. Notably, GB3‐RFP cells in the triculture condition expressed significantly higher Ki‐67 expression counted within the stromal region, compared to monoculture and coculture conditions, likely due to the presence of ECs and astrocytes (Figure 2i,j). These results indicate that the tumor expansion in the triculture condition was as a result of both invasion and increased proliferative capacity of GB3‐RFP cells within the stromal region of the platform, similar to previous studies.^[^ ^42^, ^43^, ^44^ ^]^

### 2.3 Assessment of Stem Phenotype in the Perivascular Niche

**Figure 3:**
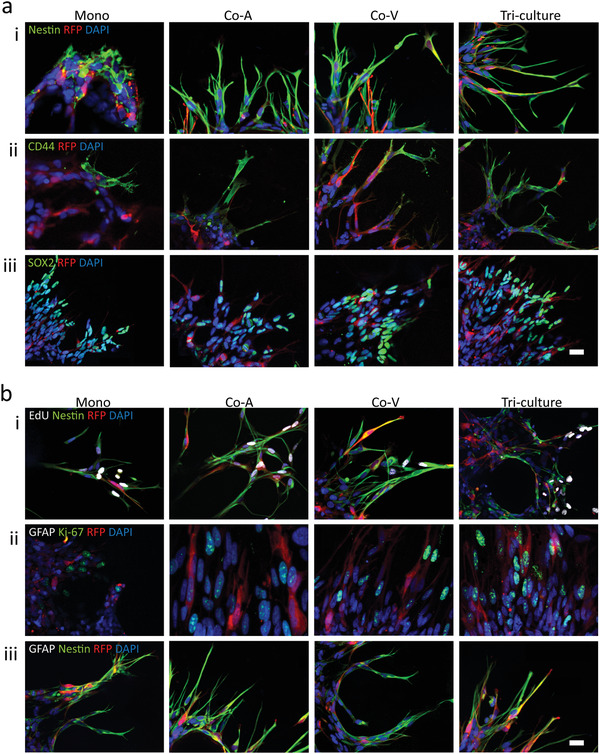
GB3‐RFP cells maintain stem phenotype within 3D microfluidic devices. a) GB3‐RFP cells demonstrated positive staining for (i) Nestin, (ii) CD44 (iii) SOX2 markers. b) GB3‐RFP cells demonstrated positive staining for proliferative markers (i) EdU when co‐stained with Nestin and (ii) negative for GFAP when co‐stained with Ki‐67. Furthermore (iii) GB3‐RFP cells co‐stained with Nestin and GFAP demonstrated positive staining for Nestin but were negative for GFAP. Scale bar: 20 µm. (Monoculture; Mono, Coculture with astrocytes; Co‐A, Co‐culture with vasculature; Co‐V)

Similar to neural stem cells, GSCs maintain their proliferation and self‐renewal in the native GBM TME.^[^ ^15^ ^]^ Astrocytes are present in large numbers in the native TME and contribute immensely to tumor progression^[^ ^45^, ^46^, ^47^ ^]^ through their role in preserving the stem‐like nature of GSCs.^[^ ^48^ ^]^ ECs also contribute to tumor progression by supporting and maintaining GSC stemness and phenotype.^[^ ^46^, ^49^ ^]^ To ensure that our platform accurately mimicked the native GBM TME, we investigated whether GB3‐RFP cells maintained their stemness in our microfluidic tumor model. Specifically, we sought to investigate the influence of astrocytes and ECs within our tumor‐on‐chip model on the functional and phenotypic properties of GB3‐RFP cells. GSCs are typically characterized through their combinatorial expression or lack of expression of specific cellular markers, albeit unexclusively.^[^ ^50^ ^]^ Expression of some putative GSC markers (e.g., CD44, Nestin, and SOX2) was investigated within our tumor PVN model. In all conditions, GB3‐RFP cells were positive for CD44, Nestin, and SOX2 (**Figure** **3**a). These results demonstrate that malignant and stemness markers are maintained in our 3D microfluidic tumor model of the GBM PVN.

We also performed EdU and Ki‐67 staining along with Nestin to determine if Nestin^+^RFP^+^ cells were proliferating. These data revealed a combination of proliferative GB3‐RFP cells and a small population which had become quiescent among all conditions (Figure 3bi,ii). GSCs, under appropriate conditions, can differentiate into cells of neuronal, astroglial, and oligodendroglial lineages in vitro. Furthermore, GSCs are also known to undergo phenotypic plasticity upon exposure to treatment or other microenvironment signals.^[^ ^51^ ^]^ To that end, in order to investigate if there was evidence of GB3‐RFP cell differentiation or plasticity, we further investigated the expression of GFAP (astrocytic and glial progenitor marker) and AQP4 (astrocyte marker). In all conditions, GB3‐RFP cells were found to be GFAP^–^ and AQP4^–^, which indicated that they were not differentiating into astrocyte lineages or demonstrating any phenotypic plasticity at least for these two markers, while astrocytes expressed GFAP and AQP4 (Figures S2 and S3a–c, Supporting Information). Under the serum‐free microfluidic tumor‐on‐chip model, GB3‐RFP cells expressed Nestin, the neural progenitor cell marker, but not GFAP, or AQP4, suggesting that GB3‐RFP cells retained their stem properties during invasion (Figure 3biii). These findings verify the contribution of key cells (ECs and astrocytes) in the TME and how they serve to maintain the stem phenotype of GSCs. Based on our data, we did not observe any differences in GB3‐RFP cells stemness markers across the four conditions, confirming that our model preserves the stem phenotype within the microfluidic device.

### 2.4 scRNA‐Seq of Cells in the Triculture Model of the Perivascular Niche

**Figure 4:**
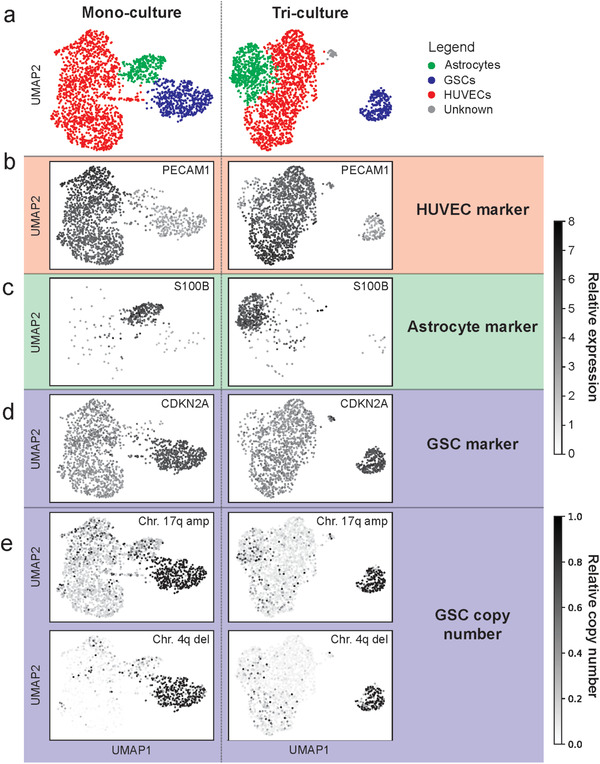
scRNA‐seq from ECs, astrocyte, and GB3‐RFP pooled monoculture and triculture. a) Using clustering of scRNA‐seq profiles and cell‐specific marker genes, we were able to identify clusters for ECs, astrocytes, and GB3‐RFP cells in pooled monoculture (left, total cells = 2109, and subpopulations ECs = 1345 cells, astrocytes = 281, and GB3‐RFP cells = 483) and triculture (right, total cells = 1947, and sub‐populations ECs = 1221, astrocytes = 477, GB3‐RFP cells = 217, and unknown = 32). b) Endothelial marker gene PECAM1 is expressed primarily in HUVECs. c) Astrocyte marker gene S100B. d) GSC marker gene CDKN2A. Copy number analysis verifies the identity of the GB3‐RFP cells via e) chromosome 17q amplification and 4q deletion (which was verified with array CGH).

To further investigate the molecular mechanisms governing the cellular integrations within the GBM PVN, we extracted the cells from the microfluidic chip model after 3 d of culture and subjected the cells to scRNA‐seq. We profiled 1947 cells from the triculture perivascular niche model (ECs = 1221, astrocytes = 477, and GB3‐RFP cells = 217) and 2109 cells from pooled mono‐culture (ECs = 1345 cells, astrocytes = 281, and GB3‐RFP cells = 483). Next, we used unsupervised cell clustering, expression of cell‐specific marker genes, and presence/absence of copy number variations (CNVs) as means to identify the three cell types in each scRNA‐seq dataset. Unsupervised cell clustering identified three and four clusters of cells for the mono‐ and triculture conditions, respectively (**Figure** **4**a; Table S2, Supporting Information). The fourth cluster of cells observed in the triculture dataset was small (*n* = 32) and it was unclear to which cell population it belonged, and therefore was excluded from downstream analysis. Subsequently, we used expression of cell‐specific marker genes to identify the three cell types: PECAM1 for ECs;^[^ ^52^ ^]^ S100B for astrocytes,^[^ ^53^ ^]^ and CDKN2A for GSCs (GB3‐RFP)^[^ ^54^ ^]^ (Figure 4b–d; Table S3, Supporting Information). GSCs were further defined by distinct copy number variants (CNVs) known to be present in the GB3‐RFP cell line based on previous CNV characterization (Figure 4e). We observed the known GB3‐RFP CNVs of Chr. 17q amplification and Chr. 4q deletion in the CDKN2A‐expressing GB3‐RFP cluster (Figure 4e), and as expected CNVs were absent in the EC and astrocyte cell clusters.^[^ ^52^ ^]^ Thus, we collected scRNA‐seq expression data for each cell type in our PVN model for both monoculture and triculture conditions.

### 2.5 Identification of Ligand–Receptor Pairs That Drive Chemotactic Invasion of GSCs

**Figure 5:**
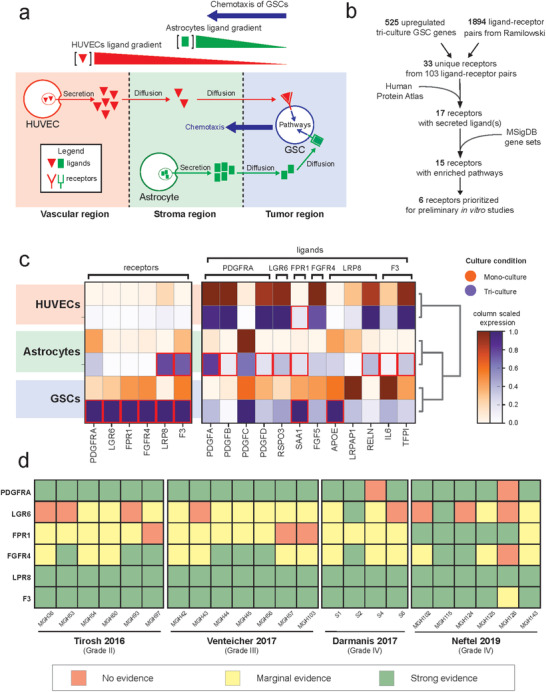
Ligand pairs are identified through scRNA‐seq and receptor expression is confirmed in neoplastic cells in primary glioma scRNA‐seq datasets. a) Project assumptions for prioritizing ligand‐receptor pairs. b) Filtering schematic to identify ligand‐receptor pairs, for more details see Experimental section. c) Matrix plots of receptor and ligand expression in the monoculture (orange) and tri‐culture (purple) conditions across cell types. Red boxes indicate significantly upregulated expression in triculture versus monoculture (Wilcoxon rank‐sum test FDR corrected *p*‐values ≤ 0.05). d) Relative receptor expression per patient across scRNA‐seq glioma datasets (*n* ≥ 113 cells per patient tumor): red = no evidence (average expression is 0); yellow = marginal evidence (average expression is greater than 0 but less than first quartile expression of all genes); green = strong evidence (average expression is greater than the first quartile expression of all genes).

We hypothesized that the increased invasiveness of GB3‐RFP cells in triculture condition is due to induced directed motility (chemotaxis) toward a gradient of ligands secreted by ECs and astrocytes (**Figure** **5**a). Therefore, we used scRNA‐seq data to prioritize receptors expressed in GB3‐RFP cells that interact with secreted ligands expressed in ECs and astrocytes (Figure 5b). First, we intersected the 525 upregulated genes in triculture GB3‐RFP cells relative to monoculture (log2(fold‐change) ≥ 0.3; FDR ≤ 0.05) with 1894 ligand‐receptor pairs^[^ ^55^ ^]^ to identify 33 upregulated receptors in triculture GB3‐RFP cells. Second, we narrowed down to 17 receptors by requiring that a secreted ligand^[^ ^56^ ^]^ must be known to interact with the receptor, and the secreted ligand must be expressed in ≥10% of either ECs, astrocytes, or both cells under triculture conditions.^[^ ^55^ ^]^ Expression of the secreted ligand SAA1 was significantly upregulated in ECs under triculture conditions relative to monoculture (annotated by red box in Figure 5c, Table S4, Supporting Information), whereas eight secreted ligands were significantly upregulated under triculture conditions in astrocytes (IL6, PDGFA, PDGFB, PDGFD, RELN, RSPO3, SAA1, and TFPI; annotated by red box in Figure 5c, Table S4, Supporting Information). Interestingly, APOE and SAA1 were also significantly upregulated under triculture conditions in GB3‐RFP, suggesting potential for autoregulatory feedback loops. Next, we refined down to 15 receptors by requiring significant enrichment of at least one canonical pathway downstream of the receptor with the genes up‐regulated in GB3‐RFP cells from triculture relative to monoculture condition (**Table** **1**, Table S5, Figure S5, Supporting information). Finally, we prioritized the six most highly upregulated receptors in the triculture GB3‐RFP relative to monoculture as candidates for downstream studies: PDGFRA, LGR6, FPR1, FGFR4, LPR8, and F3 (Figure 5c). The identification of PDGFRA as the top receptor is highly consistent with current knowledge of GBM biology as knock‐down of PDGFRA leads to decreased invasion of GSCs.^[^ ^57^, ^58^, ^59^ ^]^ Two of the most highly upregulated receptors (LGR6 and FPR1^[^ ^60^, ^61^, ^62^ ^]^) have been also implicated as drivers of invasion for other cancers, and three (FGFR4, LPR8, and F3) have little evidence of invasive properties in GBM or other cancers. Through our prioritization approach, we rediscovered and confirmed PDGFRA, a major receptor driving the chemotaxis of GB3‐RFP cells and discovered novel putative ligand‐receptor pairs.

**Table 1:** Putative ligand–receptor pairs

GSC receptor DE in triculture	log_2_(FC)H	FDR corrected *p*‐value	Ligands in ECs, astrocytes, or both	# Enriched pathways
PDGFRA	2.68	5.32 × 10^‐7^	PDGFC, PDGFA, PDGFB, PDGFD	15
LGR6	2.27	1.93 × 10^‐4^	RSPO3	1
FPR1	2.05	3.75 × 10^‐6^	SAA1	1
FGFR4	1.97	3.46 × 10^‐6^	FGF5	3
LPR8	1.94	2.23 × 10^‐7^	APOE, LRPAP1, RELN	1
F3	1.82	8.94 × 10^‐6^	IL6, TFPI	5
KDR	1.55	5.09 × 10^‐3^	SEMA6D, TIMP3, VEGFC, VEGFA, COL18A1, PDGFC	17
NOTCH3	1.50	2.81 × 10^‐3^	DLL1 DLL3, THBS2	6
PLXNA2	1.41	2.00 × 10^‐3^	SEMA6A, SEMA3A	1
CELSR1	1.30	2.18 × 10^‐2^	PSAP	1
IL6R	1.28	4.79 × 10^‐2^	IL6	7
VIPR1	1.13	2.86 × 10^‐2^	GNAS	1
SCARB1	1.08	1.89 × 10^‐2^	THBS1, SAA1, APOE	7
LDLR	0.90	2.16 × 10^‐4^	APOE, LRPAP1	10
ITGA3	0.33	1.42 × 10^‐2^	RELN, LAMB3, NID1, LAMC2, LAMC1, LAMB1, ADAM9, LAMA4, FN1, THBS1, CALR, TIMP2, LAMA5, PLAU	6

### 2.6 Expression of Prioritized Receptors in Patient Tumors and Patient Derived Cells

Next, we hypothesized that the six prioritized receptors must be expressed in at least a subpopulation of neoplastic cells in patient tumors in order to confirm their role in regulating the invasiveness of GBM tumor cells. Therefore, we analyzed the relative expression of neoplastic cells from a compendium of glioma patient tumors^[^ ^63^ ^]^, summarized the expression per tumor, and further averaged the relative expression of each receptor in the tumor. Average expression greater than zero was considered marginal evidence of receptor expression, and expression greater than the first quartile of expression was considered strong evidence of receptor expression (Figure 5d). We observed strong evidence of PDGFRA, FGFR4, LRP8, and F3 expression in all datasets. Interestingly, we found that FPR1 expression increased with tumor grade, with marginal evidence of expression in lower grade II and III gliomas and strong evidence of expression in grade IV. The receptor LGR6 expression exhibited variable expression across patients and tumor grade, suggesting an underlying heterogeneity. The presence of the six receptors in neoplastic cells from patient tumors validated the potential relevance of these receptors in the clinical setting.

To validate that the identified receptors were expressed at the protein level, we performed immunoblotting to probe for PDGFRA, LGR6, FPR1, FGFR4, LPR8, and F3 in GB3‐RFP cells grown for 72 h in conditioned media from ECs or astrocytes. We successfully detected FGFR4, PDGFRA, LGR6, and LPR8 with validated antibodies in GB3‐RFP cells (Figure S4A,B, Supporting Information). FPR1 was detected using immunofluorescence (Figure S4C, Supporting Information). Immunoblotting and immunofluorescence data further support our claim that these receptors are present in tumor cells and may drive the chemotaxis of GB3‐RFP cells towards the PVN cells in the microfluidic device.

### 2.7 Exogenous Application of Ligands Stimulates GB3‐RFP Cell Chemotactic Invasion

Next, we tested our hypothesis that the ligand–receptor interactions were driving chemotactic invasion of GB3‐RFP cells in the PVN microfluidic model. For these studies, we chose to focus on two of the most significantly upregulated and novel ligand–receptor pairs: SAA1‐FPR1, because FPR1 expression increased with grade, and RSPO3‐LGR6, because LGR6 has also been noted to be involved in various types of cancers.^[^ ^64^, ^65^, ^66^, ^67^ ^]^

**Figure 6:**
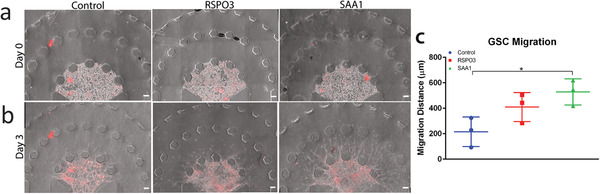
Ligand–receptor pairs are identified through scRNA‐seq and migration of GB3‐RFP cells is enhanced in response to SAA1. a) Representative images of GB3‐RFP cells on Day 0, with control condition (left). RSPO3 condition (middle) and SAA1 condition (right). b) Representative images and c) quantification of migration of monoculture of GB3‐RFPs on Day 3, revealing significantly increased migration (*p* = 0.0256) in GB3‐RFPs exposed to external SAA1 ligand than control. Data from 2 to 3 samples per experiment, over an *n* = 3 experiments. Scale bar: 100 µm.

We tested our hypothesis by adding exogenous SAA1 or RSPO3 ligand protein to the microfluidic device under GB3‐RFP monoculture conditions. Diffusion of the ligand to the tumor region recapitulated the gradient which drives chemotaxis under triculture conditions. Then we assayed for increased invasion by GB3‐RFP cells relative to vehicle control. Based on previous studies, the concentrations for SAA1 and RSPO3 were set at 15^[^ ^68^, ^69^, ^70^, ^71^ ^]^ and 1 µg mL^‐1^,^[^ ^72^, ^73^ ^]^ respectively to activate the respective pathways and study the effect on GB3‐RFP cell invasion. Three days of exposure to exogenous RSPO3 caused a marginal increase in the invasion of GB3‐RFP cells (*p*‐value of 0.108; *n* = 3; **Figure** **6**a,b). Exposure to exogenous SAA1 for the same period resulted in a significant increase in invasion GB3‐RFP cells (*p*‐value of 0.026; *n* = 3). Thus, by generating an artificial gradient of these ligands in the microfluidic device we have demonstrated that both SAA1 and to a lesser extent, RSPO3, can drive glioma tumor cell chemotactic invasion.

## 3 Discussion

The GBM TME is a complex environment composed of several cancerous and noncancerous cells, biomolecules, and ECM which synergistically contribute to tumor progression and recurrence. The GSC population has been identified as the main driving force of GBM due to the cells’ ability to evade conventional therapy and recur.^[^ ^31^ ^]^ These GSCs reside in specific niches in the TME designed to offer the optimum conditions for growth and tumor progression. Pointedly, the PVN is a pertinent element of the GBM TME that significantly influences GSC survival by promoting angiogenesis, secreting signaling cues, and engaging in bidirectional crosstalk, which ultimately shield GSCs and promote tumor progression.^[^ ^74^ ^]^ Therefore, various studies have attempted to investigate the underlying mechanisms of the PVN both in vivo and in vitro. In vivo models have been instrumental for studying disease progression within the native TME, however they cannot be utilized to dissect the causal mechanisms, within the context of cellular crosstalk, which lead to tumor invasion.^[^ ^75^, ^76^ ^]^ On the other hand, 2D in vitro models lack the physiological relevance to mimic the complexities of native niches within the local TME. Alternatively, 3D in vitro model systems, including microfluidic platforms, provide unique capabilities to properly recapitulate the complexities of the native TME^[^ ^77^, ^78^ ^]^ and to elucidate the contributions of specific components of the TME on GSCs biological behavior.^[^ ^75^ ^]^ Despite the significance, in the past few years, there have been only a few studies which have employed microfluidic models to recapitulate GBM PVN.^[^ ^79^, ^80^ ^]^ Although these studies have shed light on the influence of the PVN of the TME on tumor progression, there still remain numerous questions regarding the influence of cellular components within the PVN on GSC behavior and therapeutic resistance.^[^ ^47^ ^]^

The novelty and relevance of our work involved the development of a physiologically relevant and organotypic model of the GBM PVN. Our microfluidic platform was designed to enable integration of multiple cellular components, namely GSCs, ECs, and astrocytes to enhance its native‐like biological complexities within an organotypic architecture. Glioma cells have been shown to migrate primarily through cell‐to‐cell contacts and gap junctional communication.^[^ ^81^ ^]^ They typically exploit nerve bundles as guides and invade across nerve tracts and existing brain structures in the native TME.^[^ ^82^ ^]^ Similarly, within our developed model system, we observed high invasion and differential morphology of GB3‐RFP cells along astrocytic “migratory tracts” in the stroma region which aided in the significantly enhanced chain‐like invasion of these cells in the triculture condition consistent with previous studies.^[^ ^36^, ^38^, ^39^, ^81^, ^83^ ^]^ We further demonstrated highest migration and proliferation in our triculture platform, compared to our coculture and monoculture conditions. Various studies have also reported that both astrocytes and ECs individually increase GSCs invasion,^[^ ^45^, ^84^, ^85^ ^]^ however, we report that they may be doing so in a synergistic manner. To ensure that our 3D microfluidic model accurately recapitulated the native TME, we further confirmed that the GSCs maintained their stem fate in presence of ECs and astrocytes. Overall, our findings indicated that although the astrocytes and ECs promoted invasion of GSCs, they did not influence the stemness of these cells by causing them to acquire astroglial lineages.

Tumor niches are particularly powerful at protecting malignant cells from conventional therapies, and they provide diverse opportunities for intercellular interaction. For example, cells in the invasive GBM niche may produce and/or sense ECM, and other cells may release enzymes that act on the ECM.^[^ ^86^ ^]^ The enzymes in turn are detected by receptors on the same or different cells,^[^ ^87^ ^]^ which can trigger intracellular pathways that control other processes, such as binding to other cells or increased protein production.^[^ ^88^ ^]^ Those pathways and end‐products have their own downstream effects, which can typically be observed phenotypically. Therefore, identification of the communication molecules in and between the cells in the tumor niches will elucidate what is driving phenotypic changes. Using single‐cell RNA‐seq we identified putative ligand–receptor pairs that may be driving chemotactic behavior of GB3‐RFP cells. PDGFRA is known to be a critical gene and receptor in glioma biology.^[^ ^89^, ^90^, ^91^, ^92^ ^]^ PDGFRA abnormalities (e.g., amplification or overexpression) are currently candidates for targeted molecular therapies.^[^ ^93^, ^94^ ^]^ Therefore, capturing PDGFRA as a significantly upregulated receptor in the triculture condition, and finding all four of its ligands (PDGFA, PDGFB, PDGFC, and PDGFD) expressed in the ECs and astrocytes in tri‐culture, and enriched pathways associated with PDGFRA (BIOCARTA CBL pathway, BIOCARTA CDC42RAC pathway, KEGG MAPK signaling pathway, PID REELIN pathway, WP PI3KAKT signaling pathway, WP regulatory circuits of the STAT3 signaling pathway) supports our analyses pipeline for ligand‐receptor pair discovery in this model.

LGR6 has also been noted to be involved in various types of cancers, specifically ovarian cancer,^[^ ^64^ ^]^ skin cancer,^[^ ^65^ ^]^ gastric cancer,^[^ ^66^ ^]^ and esophageal cancer.^[^ ^67^ ^]^ Although, there has not been literature, to the best of our knowledge, that details the association of LGR6 with GBM, knockdown of LGR5 has been reported to suppress proliferation of glioma cells in vitro and in vivo.^[^ ^95^ ^]^ LGR6 had significant upregulated expression in the triculture condition and its ligand RSPO3 was present in the triculture of astrocytes, ECs, and GB3‐RFPs, although most strongly in the ECs. Additionally, of the 1289 pathways analyzed, LGR6 was mapped to one (WP GPCRS OTHER), of which was enriched in the triculture condition.

In clinical studies, increasing serum level concentrations of serum amyloid A (SAA) proteins in patients with many different types of cancer has been directly correlated with worsening tumor grade.^[^ ^96^ ^]^ Addition of SAA proteins to cancer cells in vitro has been found to stimulate metastasis formation^[^ ^70^ ^]^ and enhance invasion in certain GSC lines, while inhibiting invasion by 90% in another GSC line.^[^ ^68^ ^]^ Overall, SAA has been repeatedly identified to have a significant role in cancer migration and invasion, however the particular influence it has may be dependent on cell phenotype or culture conditions. The receptor FPR1 has been demonstrated to be a receptor for SAA1 in a cell‐based assay where it induced calcium second messenger influx.^[^ ^97^ ^]^ In this study, scRNA‐seq data revealed no expression in monoculture conditions of the SAA1 ligand nor its receptor, FPR1, in astrocytes and ECs, while GB3‐RFP cells in monoculture expressed detectable levels of SAA1 and FPR1. Moreover, in triculture conditions, expression of the ligand SAA1 from GB3‐RFP cells was greatly increased so that 100% of cells expressed the highest relative expression. Interestingly, triculture condition also induced expression of SAA1 from both astrocytes and ECs, as well as induced increased expression of FPR1 on GB3‐RFPs and downstream pathways associated with FPR1. The FPR1 receptor was found to be expressed in malignant cells from glioblastoma patient tumors. Thus, the cellular interactions that occur in triculture result in increased secretion of SAA1 from all cell types, which coincides with an increased expression of FPR1 on GB3‐RFP malignant cells, and thus the sensitivity of GB3‐RFP malignant cells to extracellular SAA1. Interestingly, SAA1 also binds the receptor SCARB1, one of the 15 up‐regulated receptors in the triculture condition driving chemotactic invasion (Figure S6, supporting information). These two ligand‐receptor pairs could explain SAA1’s increased impact over RSPO3. These findings highlight the possibility of the ligand SAA1 having a significant impact on GB3‐RFP malignant cell invasion in our model.

By investigating the intercellular interactions between the GB3‐RFP cells, astrocytes, and ECs in our PVN microfluidic model at the single‐cell level, we were able to better comprehend the underlying mechanisms driving GSC chemotaxis. By analyzing the differential gene expression in the triculture and monoculture scRNA‐seq data and implementing important filtering criteria using information from the Ramilowski ligand–receptor pair database, the Human Protein Atlas, and gene sets from canonical pathways in the Molecular Signatures Database v7.4, we discovered upregulated expression of ligand–receptor pairs in triculture, specifically, SAA1‐FPR1 and RSPO3‐LGR6, influenced by the presence of astrocytes and ECs. Most importantly, we confirmed that the ligand SAA1 significantly influenced migration of GB3‐RFPs by causing increased migration compared to control, thereby validating the scRNA‐seq analysis and ligand–receptor pair discovery pipeline. While it is unlikely that the presence of only one ligand can induce a significant migration difference between GSCs in triculture compared to other conditions, a next step could include investigation of morphology, proliferation, stemness, and migration after addition of several exogeneous ligands to GSCs grown in monoculture, or conversely, blockage of a receptor on these cells in triculture that has numerous ligands present in the ECs, astrocytes, or both. Additionally, with all the excitement surrounding cell‐to‐cell communication and its intersection with single‐cell technology lately^[^ ^98^ ^]^ updating our ligand–receptor pair reference database could be extremely beneficial. Notwithstanding, it is still reasonable to conclude that signals from astrocytes and ECs caused an expression change in the GSCs, which in turn impacted their biology and function.

## 4 Conclusion

The GBM TME has been a primary research thrust to understand how it influences tumor progression. However, the role of stromal cells like astrocytes in the TME has not been well studied. In this study, we developed a 3D microfluidic device that permits the triculture of ECs, human astrocytes, and patient‐derived GB3‐RFP GSCs. The organotypic design of our triculture model system included spatial separation of tumor, astrocytic, and vascular regions and permitted real time visualization of invading GB3‐RFP cells. Using this microfluidic platform, we investigated the migration, proliferation, and phenotypic tendencies of GB3‐RFP cells under four different experimental conditions. Our findings demonstrated that the presence of astrocytes and ECs have a synergistic effect on GB3‐RFP behavior, resulting in their increased invasive properties and elongation in their morphology. Notably, using single‐cell RNA‐seq, we identified 15 ligand–receptor pairs with upregulated receptors in GSCs, while the diffusible ligands were expressed in either astrocytes or ECs. We demonstrated that exogenous SAA1 led to significant chemotactic invasion of GSCs within our model system. Our intricate microfluidic platform presented herein is therefore a promising platform for identification of intrinsic mechanisms that promote tumor progression and future drug screening studies aimed at the GBM PVN. The proposed model can be used in a patient‐specific and high throughput manner to discover novel targets within the heterogeneous and complex GBM TME as already demonstrated in our work. Our future studies will employ immune cells to study their contribution to chemoresistance and immunosuppression within the GBM TME.

## 5 Experimental Section

### Microfluidic Design and Fabrication

The microfluidic platform was designed using AutoCAD software and printed onto a transparent mask. The design consisted of an inner tumor region bordered by two concentric semicircles serving as the stroma and vascular regions. The diameters of these regions were 1, 2.5, and 3.5 mm, respectively. The tumor and stromal regions were bound by hexagonal microposts spaced evenly at 100 µm, while the vascular region was bound externally by trapezoidal microposts spaced evenly at 100 µm. The hexagonal design was present in boundaries that were flanked by gel regions, as they prevented leakage of hydrogel by increasing the contact angle of the gel and the hexagonal microposts, thereby reducing the built‐up internal pressure acquired during gel injection. The spacing of the microposts enabled crosstalk between adjacent regions and diffusion of media and biomolecules throughout the platform, while providing spatial separation between regions. Using SU8‐2075 (MicroChem) photolithography technology, a master mold was created by spinning to a height of 200 µm onto a 4 in. silicon wafer, after which the wafer and the transparent mask with device designs were exposed to ultraviolet (UV) radiation to form a primary mold. After development of the wafer, it was treated with methyltrichlorosane (MTCS, Sigma‐Aldrich) to render the surface of the wafer hydrophobic to ensure easy retraction of the polydimethylsiloxane (PDMS, Slygard 184 Silicon Elastomer Kit, Dow Corning). PDMS was mixed at a 10:1 ratio of base to curing agent, poured on the wafer, degassed, then cured for 1.5–2 h at 80 °C. The PDMS cast was then peeled from the mold and punched with biopsy punches to create inlets and outlets. Individual devices were then cut from the PDMS cast and bonded to 18 mm^2^ coverslips to form microfluidic channels. Before bonding, the PDMS casts and coverslips were wiped with ethanol and pressurized nitrogen gas to rid all particles and dirt, after which they were exposed to oxygen plasma (PDC‐32G, Harrick Plasma) to ensure hydrophilicity. The PDMS casts were then bonded face down onto the coverslips with slight pressure to ensure attachment. Following, the bonded devices were placed in an 80 °C oven overnight to secure the bonds. To ensure that devices were sterilized before use, they were subsequently placed in a liquid and dry autoclave, after which they were placed in an 80 °C oven and allowed to dry overnight.

To aid in the attachment of hydrogels (i.e., Fibrin and Matrigel) to specific regions (i.e., tumor, stroma, vascular), devices were treated with poly‐d‐lysine (1 mg mL^‐1^) (PDL, Sigma‐Aldrich) followed by glutaraldehyde (1% (v/v)) (Sigma‐Aldrich). Specifically, PDL was injected into the designated regions of the device and incubated at 37 °C for 1 h, after which the devices were washed once with deionized (DI) water. Glutaraldehyde was then introduced into the regions, and devices were incubated at room temperature for 1.5–2 h, after which they were washed five times with DI water to remove excess glutaraldehyde. Devices were then left in an 80 °C oven overnight to restore hydrophobicity and allow microposts to contain hydrogels before polymerization.

### Cell Culture

GSC patient samples used for this research were provided by the Biobank Core Facility at St. Joseph's Hospital and Medical Center and Barrow Neurological Institute (BNI), Phoenix, Arizona. The samples were innominate and followed the Biobank Institutional Review Board (IRB) protocol. Patient‐derived cell line, GB3, was established based on previous protocols.^[^ ^15^ ^]^ Briefly, tumor tissue was processed using a Tumor Tissue Dissociation kit (Miltenyi Biotech Inc.), and cells were expanded as spheroids in neural stem cell (NSC) medium comprising Dulbecco's modified Eagle media (DMEM) and F12‐Glutamax, supplemented with N2, B27, and Pen‐strep (Fisher Science). Cells were spiked with 20 ng mL^‐1^ epidermal growth factor (EGF) and 20 ng mL^‐1^ fibroblast growth factor (FGF, EMD Millipore) every other day until confluent. To create a GB3RFP cell line, GB3RFP cells were transduced with already‐made lentiviral particles (Amsbio) expressing RFP‐Luc. To maintain high transduction efficiency of the GB3‐RFP cell line, blasticidin (2 µg mL^‐1^) was added to cell culture media every time the cells were passaged. Use of GB3‐RFP cells for experiments was discontinued after the cells reached passage 30.

Human astrocytes (Mehta lab, BNI) were grown in astrocyte basal medium supplemented with N2 and 10% fetal bovine serum (FBS) (Glutamax, Fisher Science). Media was changed every other day, and cells were only used between passages 4–10. Astrocytes were used for 3D cultures at 70–80% confluency.

Human umbilical vein endothelial cells (HUVEC, Lonza) were cultured in Endothelial Growth Medium (EGM‐2, Lonza). Media was changed every other day, and cells were used at 70–80% confluency. HUVECs were only used between passages 3– 7, and all cells were grown under standard conditions (humidified, 37 °C, 5% CO_2_).

### Vasculogenesis in the Microfluidic Device

For vasculogenesis within the tumor model, ECs at 70–80% confluency were primarily dissociated from tissue culture flasks using trypsin–EDTA. To make the fibrinogen solution, bovine fibrinogen (Sigma‐Aldrich) was dissolved in Dulbecco's phosphate‐buffered saline (DPBS, Gibco) at 5 mg mL^‐1^. Afterward, bovine thrombin (Sigma‐Aldrich) was dissolved in DPBS to form a 4 U mL^‐1^ thrombin solution. The fibrinogen and thrombin solutions were stored at ‐20 °C after filter sterilization (Denville Scientific) to prevent denaturing. ECs, fibrin, and thrombin were mixed in a 1:1:1 ratio to form a hydrogel with a final density of 20 × 10^6^ cells mL^‐1^, similar to our previous study.^[^ ^15^ ^]^ The mixture was immediately injected into the vascular region of the device. All solutions were kept on ice to avoid premature gel polymerization. After injection, devices were incubated for 10 min at 37 °C to encourage fibrin polymerization and flipped every minute to ensure even distribution of cells in the 3D matrix. Next, EGM‐2 supplemented with 50 ng mL^‐1^ vascular endothelial growth factor (VEGF, Millipore) was added to the media channels of the devices. The devices were then placed in a larger petri dish containing DI water, to provide extra humidity to prevent media evaporation in devices, and incubated at standard conditions (humidified, 37 °C, 5% CO_2_) for 72 h. Cell culture media was exchanged with EGM‐2 dosed with VEGF every 24 hours throughout vasculogenesis (3 d).

### Establishment of GB3‐RFP Cell Invasion Assay within the Tumor‐on‐Chip Platform

To form triculture within microfluidic tumor‐on‐chip platform and study GSC invasion, upon formation of vascular network within the third region of the platform, GB3‐RFP cells and astrocytes were injected into the tumor and stroma regions of the device, respectively. Specifically, GB3‐RFP spheroids were dissociated using Accutase (Invitrogen). The cell suspension was mixed with an equal portion of Matrigel (Corning) in a 1:1 ratio to form a final density of 15 × 10^6^ cells mL^‐1^. The cell‐hydrogel solution was then immediately injected into the tumor region of the device and allowed to polymerize at 37 °C for 4–5 min. Devices were flipped every minute to ensure even distribution of cells within the gel. After polymerization, astrocytes dissociated with trypsin–EDTA were suspended in pure Matrigel in a 1:1 ratio to form a final density of 3 × 10^6^ cells mL^‐1^. The cell‐hydrogel solution was then immediately injected into the stroma region of the devices very carefully to avoid leakage into adjacent regions. Devices were allowed to polymerize at 37 °C for 5–6 min while being flipped every minute. NSC media was then added to the media channels. For coculture conditions involving GB3‐RFP cells and astrocytes, GB3‐RFP cells and astrocytes embedded in pure Matrigel were incorporated into the tumor and stroma regions respectively. Specifically, GB3‐RFP cells dissociated with Accutase (Invitrogen) were mixed with an equal portion of Matrigel (Corning) in a 1:1 ratio to form a final density of 15 × 10^6^ cells mL^‐1^. The cell‐hydrogel solution was then immediately injected into the tumor region of the device and allowed to polymerize at 37 °C for 4–5 min while flipping. Following, astrocytes were suspended in pure Matrigel in a 1:1 ratio to form a final density of 3 × 10^6^ cells mL^‐1^. The cell‐hydrogel solution was then immediately injected into the stroma region of devices and allowed to polymerize at 37 °C for 5–6 min, while being flipped every minute. Pure Matrigel was then injected into the vascular region and allowed to polymerize at 37 °C for 3 min. Next, NSC media was added to the media channels, and devices were incubated under standard conditions for 72 h. For coculture of EC's and GB3‐RFP cells, GB3‐RFP cells suspended in pure Matrigel at a final density of 15 × 10^6^ cells mL^‐1^ were embedded into the tumor region upon formation of vascular networks in the vascular region. The GB3‐RFP cell‐hydrogel solution was allowed to polymerize for 4–5 min in the device while flipping. After polymerization, pure Matrigel was injected into the stroma region to allow successful diffusion of nutrients from the media channels to the tumor region. NSC media was added to the media channels, after which devices were kept under standard conditions. Finally, to establish monoculture of GB3‐RFP condition, GB3‐RFP cells were suspended in pure Matrigel at a final density of 15 × 10^6^ cells mL^‐1^ and allowed to polymerize for 4–5 min. Following, pure Matrigel was injected into the stroma and vascular regions, after which NSC media was added to the media channels. Devices were then kept in the incubator under standard conditions for 72 h and media was changed daily in all conditions.

### GB3‐RFP Cell Invasion Analysis

To characterize the invasion pattern of GB3‐RFP cells, phase‐contrast images were captured 72 h after GB3‐RFP cell insertion and invasion distance was measured by drawing lines from the microposts of the tumor region to the edge of the leading cell extensions, and then averaging at least 10 lines. To characterize the morphology of the GB3‐RFP cells across the four experimental conditions, three statistical measures were employed: nuclei per chain, nuclei per field of view, and extension from cell body. To measure nuclei per chain, the number of nuclei per GB3‐RFP cell extension was counted and compared. To determine nuclei per field of view, the number of nuclei in five fields of view was considered. Finally, extension per cell body involved measuring the length of the GB3‐RFP cell extension from the edge of the nucleus to the end of its extension and comparing across experimental conditions.

### Immunofluorescence Staining

For immunofluorescence (IF) staining, cells in the microfluidic devices were fixed with warmed 4% paraformaldehyde (PFA) by removing the cell culture medium and replacing it with PFA, following a PBS wash. Devices were incubated at room temperature for 30 min, after which devices were washed twice with PBS–glycine (100 × 10^‐3^ m glycine in PBS), and once with PBS‐Tween (0.05% (v/v) Tween‐20 in PBS) for 10 min each at room temperature. To permeabilize the cells, IF buffer (0.2% (v/v) Triton X‐100, 0.1% (v/v) BSA (radioimmunoassay grade), 0.05% Tween 20, 7.7 × 10^‐3^ m NaN_3_ in PBS) was added to the outlets of the devices and negative pressure was applied to the inlets to create flow. The devices were then incubated at room temperature for 30 min. Following this, to block the cells, 10% goat serum in PBS‐Tween was added to the devices in similar fashion, and devices were incubated for an additional hour to prevent nonspecific binding of antibodies.

Next, primary antibodies of interest were diluted in goat serum and added to the devices. The samples were then placed in petri dishes and taped with parafilm to prevent evaporation. The devices were kept at 4 °C overnight to ensure thoroughly targeted binding of antibodies. After exposure to primary antibodies, devices were washed three times each at 20 min intervals with PBS‐Tween at room temperature. Alexa Flour‐conjugated species matching secondary antibodies (1:500) diluted in PBS‐Tween were centrifuged at 14k RPM for 10 min and added to devices. Devices were incubated for 45 min–2 h in the dark. They were then washed 3–5 times in PBS‐Tween at 20 min intervals each, after which 4’6‐diamidino‐2‐phenylindole (DAPI, Invitrogen) at a 1:1000 dilution was added to the devices and kept at 4 °C overnight. Next, devices were finally washed five times at 10 min intervals in PBS‐Tween.

The antibodies used were anti‐Human Nestin (1:200, (10C2) Mouse NB300‐266 (Novus Biologicals)), anti‐GFAP (1:400, (GA5) Mouse mAb #3670 (Cell Signaling Technologies)), anti‐SOX2 (1:400, (D6D9) XP Rabbit mAb #3579, (Cell Signaling Technologies)), anti‐Ki‐67 (1:1000, Rabbit ab15580 (Abcam)), mouse CD31 (10 µg mL^‐1^, (P2B1) (Developmental Studies Hybridoma Bank)).

### Fibrinolysis and Cell Extraction from the Platform

For subsequent single‐cell RNA‐sequencing (scRNA‐seq) analyses, ECs, astrocytes, and GB3‐RFPs were cultured individually in microfluidic devices and in a triculture fashion as previously described. Cells were extracted from microfluidic devices using collagenase type II (2 mg mL^‐1^, Thermo Fisher Scientific) and/or Nattokinase (70 FU, NSKD, Japan Bio Science Ltd). Specifically, GB3‐RFP monoculture and astrocyte monoculture devices were digested with collagenase type II, while EC monoculture and GB3‐RFP‐astrocyte‐EC tri‐culture devices were digested with both collagenase type II and Nattokinase due to presence of a vascular network. Collagenase type II was diluted in PBS containing 1 × 10^‐3^ m EDTA at a concentration of 2 mg mL^‐1^. Nattokinase was also diluted in 1 × 10^‐3^ m EDTA and used at an activity of 70 fibrinolytic units (FU). Briefly, devices were washed with PBS once, after which PBS was exchanged for either collagenase type II solution or Nattokinase. For conditions involving collagenase, the devices were incubated for 15–30 min at room temperature. Following incubation, physical agitation was used to dissociate and collect cells from the device. Devices were washed twice with PBS, and the final cell suspension was centrifuged. After centrifugation, the supernatant was gently aspirated, and cells were washed once with PBS containing 0.04% w/v BSA. 20 µL of the total cell suspension was reserved for cell counting and viability measurements. The total cell suspension was immediately recentrifuged and resuspended in PBS containing 0.04% w/v BSA at a final concentration of 1100 cells µL^‐1^. For the triculture condition, devices were washed once with PBS, after which Nattokinase was added to the samples and incubated for 15 min at room temperature. Following incubation, ECs were physically agitated to ensure dissociation. Devices were then washed twice with PBS, and the resulting EC suspension was centrifuged. The supernatant was then aspirated and replaced with PBS containing 0.04% w/v BSA. Next, collagenase type II solution was added to the same devices and incubated for an extra 30 min. After physical agitation, astrocytes and GB3‐RFP cells were collected from the devices. The devices were then washed twice with PBS to collect any remaining cells and the final cell suspension was centrifuged. After centrifugation, the supernatant was aspirated, and the cells were resuspended in PBS containing 0.04% w/v BSA. The resulting cell suspension was then mixed with the ECs and recentrifuged. The cells (ECs, astrocytes, and GB3‐RFP) were then resuspended in PBS containing 0.04% w/v BSA at a final concentration of 1100 cells µL^‐1^.

### Single‐Cell RNA Sequencing (scRNA‐Seq) Sample Preparation

scRNA‐seq was applied to both monoculture of each cell type and triculture of all three cell types in the microfluidic model. For the monoculture conditions, each cell line (GB3‐RFP, ECs, and astrocytes) was cultured separately in their respective regions of the device (i.e., tumor, stroma, vascular). Cells were extracted after 72 h and pooled at 1:1:1 ratio for scRNA‐seq profiling, and the cell type was deconvoluted post‐hoc as described later. The triculture condition involved culturing of all three cell types in the same microfluidic device. Cells were extracted after 72 h of coculturing. Cell density was titrated to characterize approximately 2600 single cells. scRNA‐seq libraries were prepared using 10X Genomics Chromium Next Gem Single Cell 3’ Kit v3.1. The quality of each library was determined using Agilent TapeStation automated electrophoresis. Libraries were sequenced using Illumina HiSeq with approximately 100 000 reads per cell. The 10X Genomics CellRanger v4.0.0 was used to align to the human reference genome GRCh38‐2020‐A, quantify, and provide basic quality control metrics for the scRNA‐seq data.

### Cell Clustering and Identification of Marker genes

scRNA‐seq data from the pooled monoculture and triculture conditions were analyzed using Scanpy 1.8.2. Data were loaded as counts, normalized, and scaled while considering both percent mitochondria and the number of UMIs per cell as covariates. Initial filtering removed genes present in less than 3 cells, cells with less than 200 genes, and cells with mitochondrial RNA greater than 18% of total RNA. Data were filtered to the top 4000 variable genes before principal component analysis (PCA), and clusters of cells were identified using a shared nearest neighbor (SNN) modularity optimization‐based clustering algorithm. Marker genes for each cluster were identified as differentially expressed genes (‐0.3 ≥ log2(FC) ≥ 0.3; FDR ≤ 0.05), and the determination of 3 and 4 cell types for mono‐ and triculture, respectively, were based on the discovery of strong marker genes for three of the clusters (triculture data had an unknown cluster of a few cells). Marker genes included PECAM1^[^ ^52^ ^]^ for ECs, S100B^[^ ^53^ ^]^ for astrocytes, and CDKN2A^[^ ^54^ ^]^ for GB3‐RFP cells.

### Identification of Ligand–Receptor Pair Transcript Expression in Triculture

Mono‐ and triculture scRNA‐seq data were integrated, subset to the common 20457 genes and normalized. Differentially expressed genes between mono‐ and triculture of the same cell type were then computed. The rank_gene_groups function was used to identify 525 genes upregulated in the GB3‐RFP cells from the triculture condition compared to the GB3‐RFP cells cultured independently (monoculture) (log2(FC) ≥ 0.3; FDR ≤ 0.05). A network of 1894 literature‐supported ligand–receptor interactions was used to identify putative receptor genes among the differentially expressed triculture GB3‐RFP genes.^[^ ^55^ ^]^ The receptor genes were also required to have at least one literature‐supporting interaction with a secreted ligand,^[^ ^56^ ^]^ of which that ligand was present (expression in ≥10% cells) in ECs or astrocyte cells from the triculture condition.

Next, pathways were identified where the receptor gene was included from the Molecular Signatures Databse (MSigDB), specifically, BIOCARTA, KEGG, GO Biological Processes, WikiPathways (WP), and NCI‐PID (PID). These pathways were then used for significant enrichment with genes up‐regulated in the GB3‐RFP cells grown in triculture versus monoculture (log2(FC) ≥ 1.0; FDR ≤ 0.05) using hypergeometric enrichment scores calculated using the scipy.stats hypergeom.sf function. Receptors with pathways that had hypergeometric enrichment *p*‐values ≤0.05 were considered to have significant downstream pathway activation.

The gene sets of each pathway were summarized into a single vector using the first principal component corrected for sign, which is referred to as the eigengene. Eigengenes were calculated for 128 curated gene sets derived from the BioCarta, KEGG, PID and WikiPathways pathway databases and mapped to 15 unique receptor genes.

### Expression of Putative GB3‐RFP Receptors in Patient scRNA‐Seq Glioma Datasets

Relative receptor expression was obtained as described in ref. [63] from four glioma primary patient tumor scRNA‐seq studies: i) grade II oligodendrogliomas that are IDH1 mutant from ref. [99]—GSE70630; ii) grade III astrocytoma's that are IDH1 mutant from ref. [100]—GSE89567; grade IV glioblastomas that are IDH wild type from ref. [101]—GSE84465; and iv) grade IV glioblastomas that are IDH wild type from ref. [102]—GSE131928. For each dataset, raw count data were used and input into Seurat V3 for basic filtering, normalization, scaling, clustering, etc. Meta‐data were loaded into R statistical software and imported into the Seurat object for later filtering and comparison. Neoplastic cells were enriched for by removing cells from de novo clusters marked by terminally differentiated cell type genes (e.g., MBP, PLP1), immune cells (e.g., CD14, AIF1), astrocytes (ETNPPL), etc. The processed Seurat object was saved as a loom file for loading ease into python and downstream expression analyses. In python, data were split according to patient, and cells were subset and normalized to both i) only the receptor of interest and ii) all the genes that had expression in at least 3 cells. The average expression and first quartile expression were tabulated for the two cell subsets, respectively, and compared between each other.

### Conditioned Media Preparation

ECs, astrocytes, and GB3‐RFP cells were cultured in their respective standard culture media until ≈60% confluency. The culture vessels were washed twice with PBS 1× and media was changed to NSC media. After 24 h, the conditioned media was collected and syringe filtered with a 0.2 µm filter, and stored at ‐80 °C.

### Western Blots of Receptors on GB3‐RFP Cells Incubated with Conditioned Media

GB3‐RFP cells were plated at a density of 150 000 cells per well on six‐well laminin‐coated tissue culture plates overnight. The next day, the neural stem cell media was replaced with respective conditioned media (EC conditioned media or astrocyte conditioned media). Cells were homogenized after 72 h in RIPA lysis buffer containing protease and phosphatase inhibitors (ThermoFisher Scientific), rotated at 4 °C for 20 min and then centrifuged at 15 000 RPM for 10 min at 4 °C. Protein concentrations from whole‐cell extracts were determined using the Bradford Protein Assay (ThermoFisher Scientific). Equal amounts of protein (30 µg per lane) were loaded onto a 7.5% 10% or 12.5% SDS‐PAGE gels and transferred to a polyvinylidene fluoride membrane (PVDF; Millipore‐Sigma).

Membranes were blocked with 5% nonfat milk for 1 h at room temperature and incubated overnight with primary antibodies at 4 °C; Primary antibodies used in this study were mouse antimouse anti‐*β*‐actin (1:1000, Bio‐Rad, MCA5775GA), mouse anti‐Vinculin (1:10 000, Millipore 05‐386), rabbit anti‐LGR6 (1:1000, Abcam, ab126747), rabbit anti‐LRP8 (1:1000; Abcam, ab108208). Membranes were probed with fluorophore‐conjugated antimouse or antirabbit secondary antibodies (1:10 000; ThermoFisher Scientific). Western blots were developed using the LI‐COR Odyssey CLx imaging system (LI‐COR Inc.) and quantitated using the Image Studio Lite software. All Western blots are representative images from a minimum of three biological replicates.

### Stimulation of Expressed Receptor in GB3‐RFP Cells in Monoculture to Study Influence on Migration

Exogenous ligand protein was added to the media channel in the microfluidic device and allowed to diffuse to the tumor region to recapitulate the gradient which drives chemotaxis in triculture conditions. Fibrin was used to fill the vascular layer in experiments that involved addition of external proteins to the media channels. Briefly, fibrinogen (5 mg mL^‐1^) and thrombin (4 U mL^‐1^) were mixed 1:1, and immediately injected into the vascular region of the device. The device was incubated for 10 min at 37 °C. It is confirmed that GSC invasion was not influenced by the type of gel injected into the vascular region (i.e., monoculture with fibrin vs monoculture with Matrigel) (Figure S7, Supporting Information). GB3‐RFP cells were dissociated with Accutase, then resuspended in NSC media at a concentration of 60 × 10^6^ cells mL^‐1^. The GB3‐RFP cells were mixed 1:1 with Matrigel, to render a final concentration of 30 × 10^6^ cells mL^‐1^, and immediately injected into the tumor region. The devices were incubated at 37 °C for 5 min, during which they were flipped every minute to ensure 3D tissue formation. Then, Matrigel was injected into the stromal region, and the devices were incubated for another 6–7 min at 37 °C. Finally, NSC media was added to the media channels for the control groups, while the ligand from the prioritized ligand–receptor pairs identified from scRNA‐seq filtering pipeline (either SAA1 at 15 µg mL^‐1^ or RSPO3 at 1 µg mL^‐1^) was resuspended in NSC media and added to the media channels on the device, for their respective experimental groups. Media was changed daily with the designated media formulation, and the devices were imaged and fixed on day 3 to assess GSC invasion distance.

### Imaging

Microfluidic devices were imaged with fluorescence microscopy (Zeiss Axio Observer ZI with Zen Pro software suite) equipped with Apotome.2 (Zeiss) at 10× and 20× magnification. Phase contrast images were uploaded into NIH ImageJ software (NIH ImageJ software) and processed, while fluorescent images were processed in both ImageJ and Zen Pro Imaging software. Besides mono‐cultured ECs which were captured in the vascular region, all IF images were captured within the stroma layer of the device.

### Statistical Analysis

For all experimental analyses, values were obtained from at least three independent experiments (*n* > 3) with at least three technical replicates each. Reported measurements were expressed as average ± standard deviation. The data were compared using Two‐way ANOVA with Tukey's multiple comparison test. Statistical analysis was performed using GraphPad Prism software (GraphPad Prism 9.2).

## Conflict of Interest

The authors declare no conflict of interest.

## Supporting information

Supporting Information

Supporting Table 1

Supporting Table 2

Supporting Table 3

Supporting Table 4

## Data Availability

The data that support the findings of this study are available in the supplementary material of this article.
