# Supplementary material for: Investigating the Interactions of Glioma Stem Cells in the Perivascular Niche at Single‐Cell Resolution using a Microfluidic Tumor Microenvironment Model
Source: Adv Sci (Weinh). 2022 May 26;9(21):2201436. doi: 10.1002/advs.202201436 (PMC9313491; doi:10.1002/advs.202201436)
Supplement: Supplementary file 1 — Supporting Information [file ADVS-9-2201436-s003.pdf]

ADVANCED SCIENCE
Open Access

Supporting Information

for *Adv. Sci.*, DOI 10.1002/advs.202201436

Investigating the Interactions of Glioma Stem Cells in the Perivascular Niche at Single-Cell Resolution using a Microfluidic Tumor Microenvironment Model

Emmanuela A. Adjei-Sowah, Samantha A. O'Connor, Jaimeson Veldhuizen, Costanza Lo Cascio, Christopher Plaisier, Shwetal Mehta and Mehdi Nikkhah**

Investigating the Interactions of Glioma Stem Cells in the Perivascular Niche at Single-Cell Resolution using a Microfluidic Tumor Microenvironment Model

Emmanuela A Adjei-Sowah[1#], Samantha A O'Connor[1#], Jaimeson Veldhuizen[1#], Costanza Lo Cascio[3], Christopher Plaisier[1], Shwetal Mehta[3*], and Mehdi Nikkhah[1,2*]

[1]School of Biological and Health Systems Engineering, Arizona State University, Tempe, AZ 85287-9709, USA

[2]Virginia G. Piper Biodesign Center for Personalized Diagnostics, Arizona State University, Tempe, AZ 85287-9709, USA

[3]Ivy Brain Tumor Center, Barrow Neurological Institute, St. Joseph's Hospital and Medical Center, Phoenix, AZ, 85013

#These authors contributed equally to this work.

*CORRESPONDING AUTHORS:

Mehdi Nikkhah (mnikkhah@asu.edu)

Shwetal Mehta (smehta@barrowneuro.org)

SUPPLEMENTARY INFORMATION

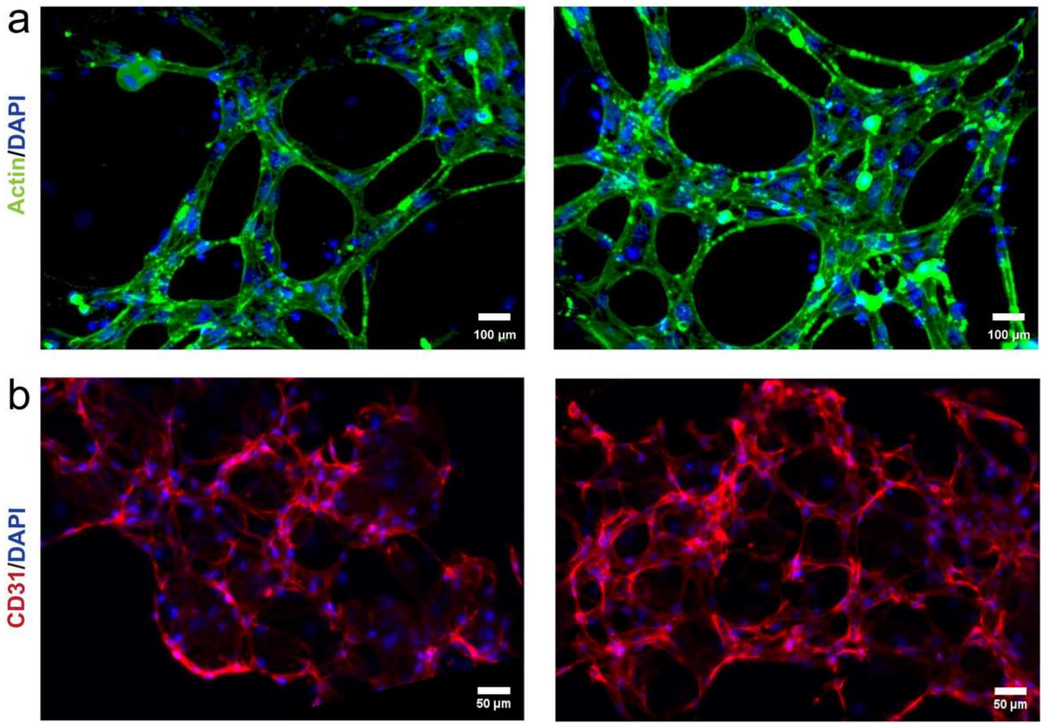

Supplementary Figure 1: Representative F-actin and CD31 images, demonstrating network formation and interconnectivity of the vessels within the vascular region of the microfluidic tumor model.

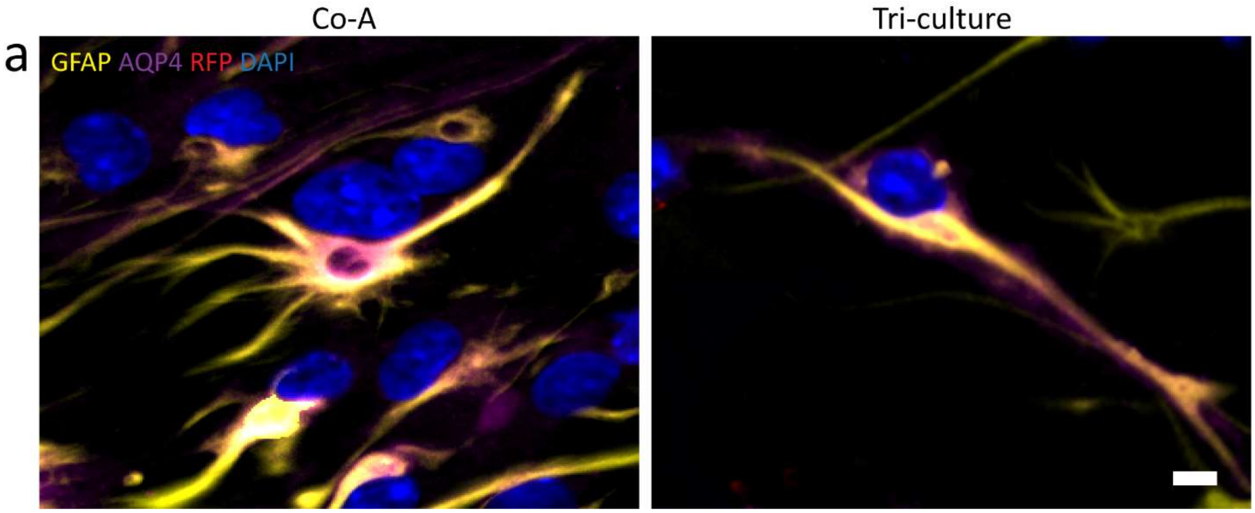

Supplementary Figure 2. **Astrocytes express GFAP and AQP4** in (A) Co-culture with astrocyte condition (Co-A) and (B) Tri-culture condition. Scale bar: 50 μm.

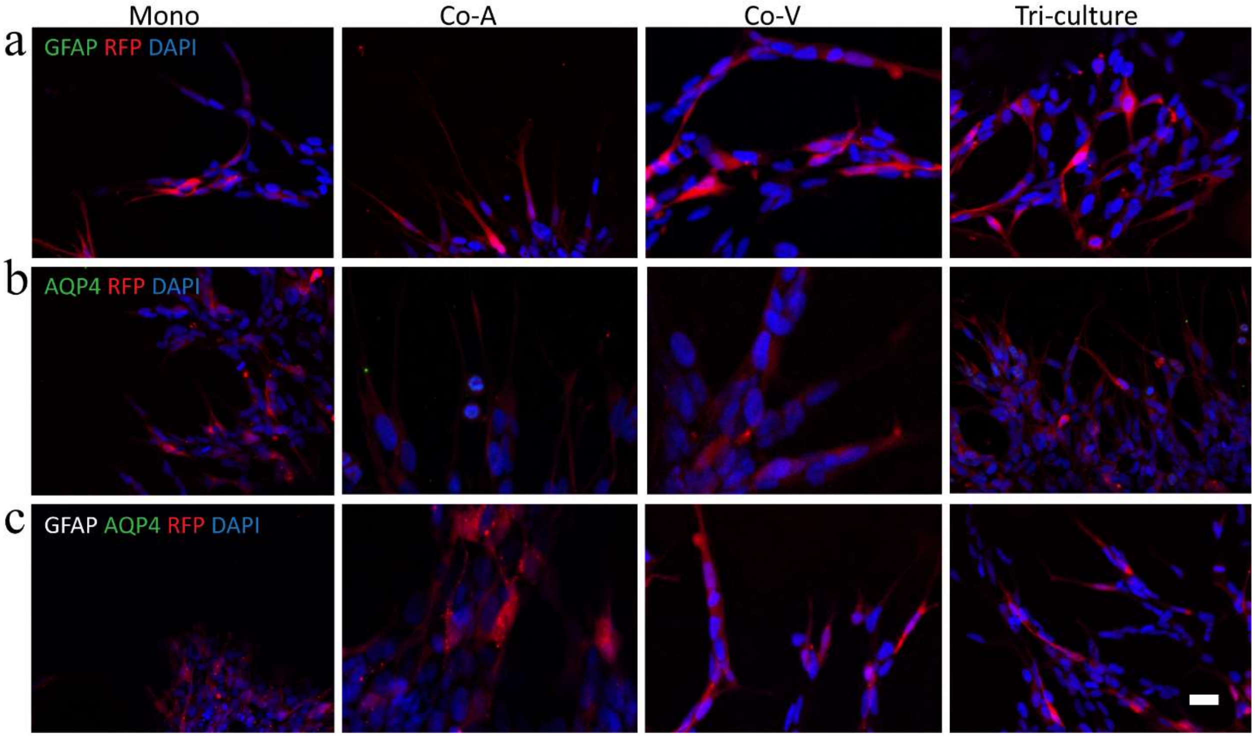

Supplementary Figure 3. GB3-RFP Glioma Stem do not differentiate within 3D microfluidic devices. GB3-RFP cells demonstrate negative expression for (**A**) GFAP and (**B**) AQP4 (**C**) and GFAP + AGP4 co-stain. (Co-A: Co-culture with astrocytes, Co-V: Co-culture with vasculature). Scale bar: 20 µm.

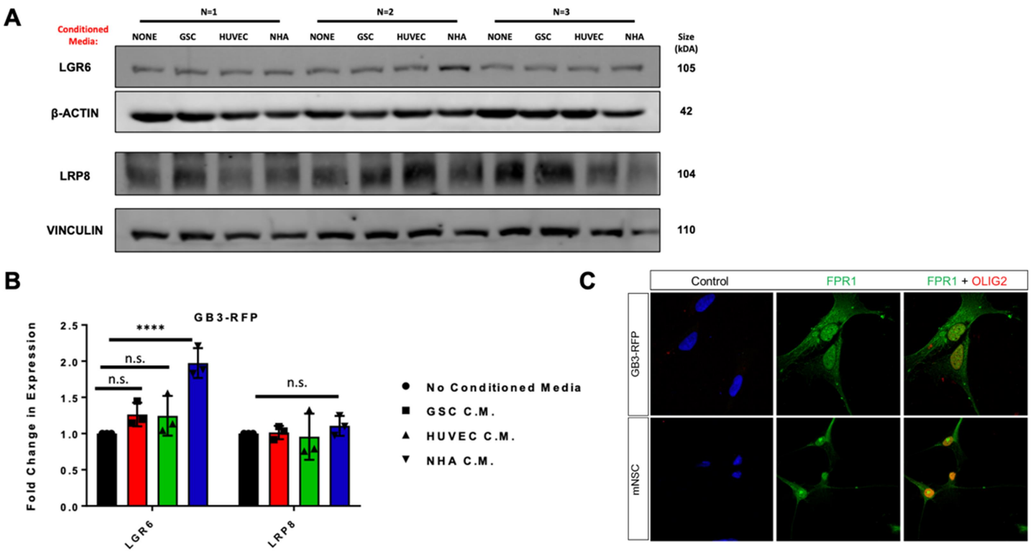

Supplementary Figure 4. **Protein expression of LGR6 and LRP8. A.** Expression of LGR6 and LRP8 in GB3-RFP cells cultured in presence of GSC media or conditioned media from GSC, HUVEC, or NHA (astrocytes) cells. **B.** Quantification of fold change in LGR6 and LRP8 expression in presence of conditioned media. **C.** Immunofluorescence analysis of FPR1 (green) and OLIG2 (red) expression in GB3-RFP and murine neural stem cells (mNSCs). Nuclei are stained with DAPI. (**** < 0.0001; n.s. = not significant; n=3 for each data set).

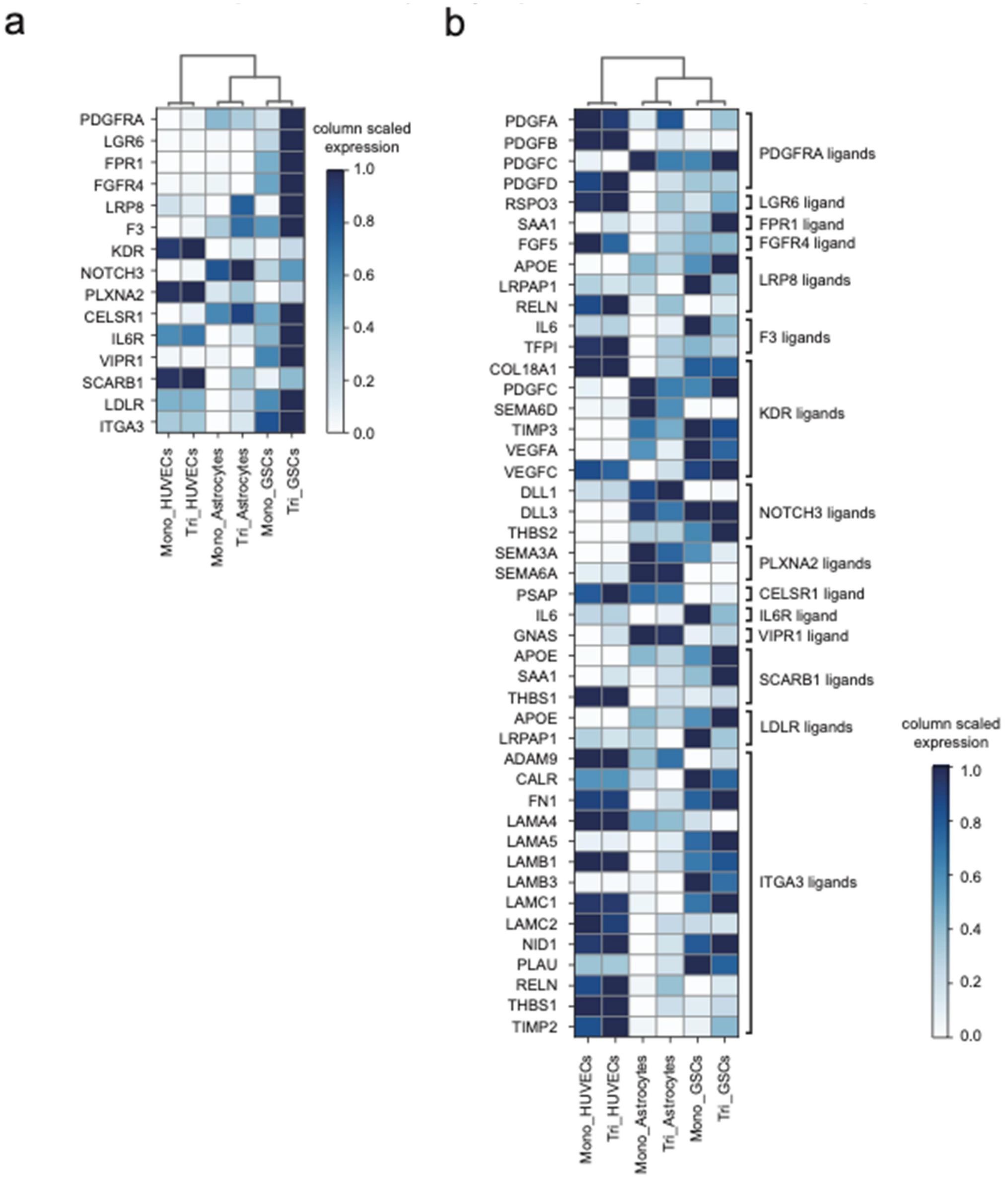

Supplementary Figure 5. Receptors that passed al filters. A) Average expression of 15 receptors that passed all filters. B) Average expression of ligands known to bind receptors in A.

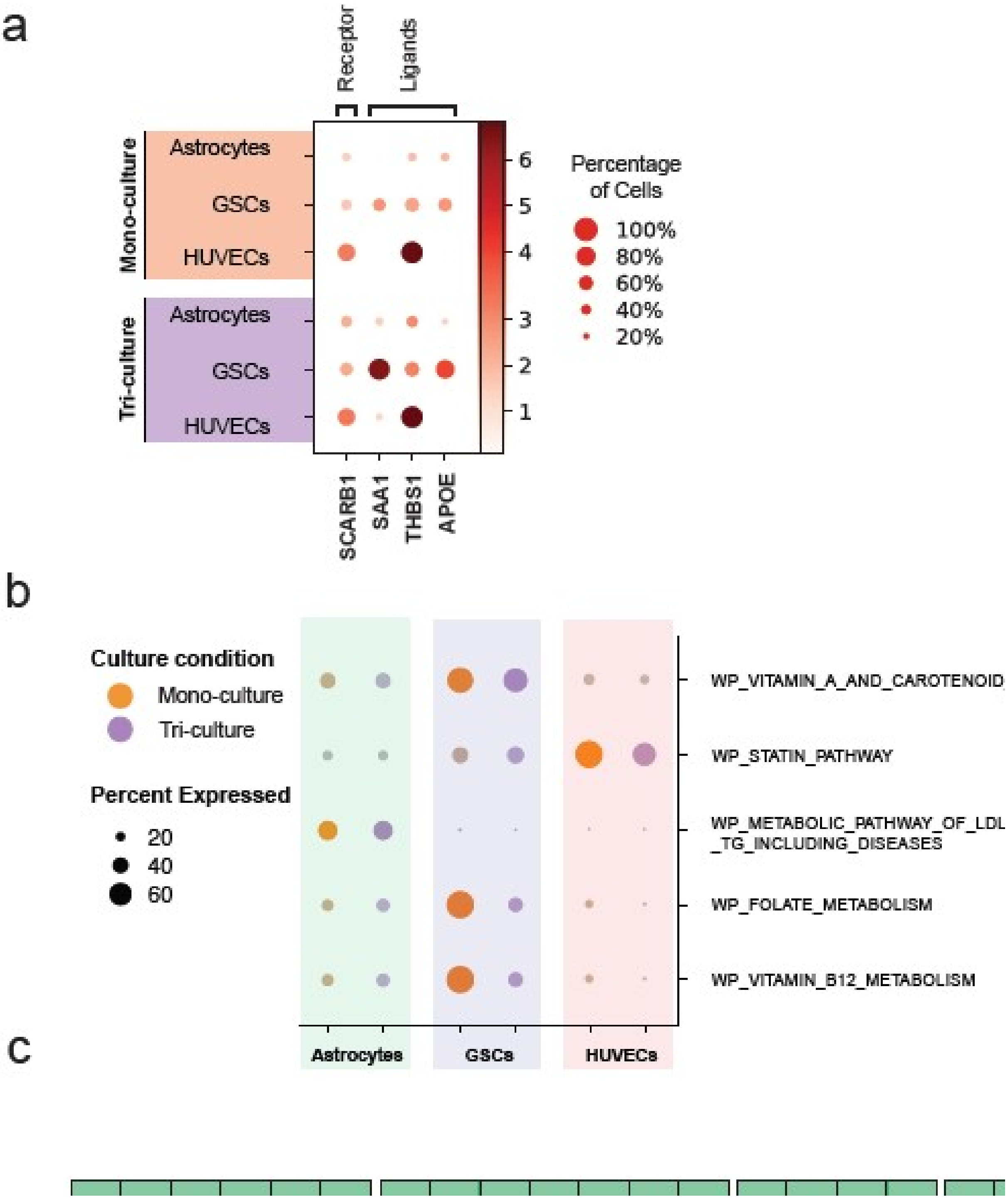

Supplemental Figure 6. A) Expression of SCARB1, SAA1, THBS1, APOE in each cell type in each condition. B) Eigengene expression of enriched pathways associated with SCARB1 plotted for each cell type in each condition C) Expression of SCARB1 in primary scRNA-seq glioma datasets. Mean SCARB1 expression was greater than the first quartile (and average) expression of all genes for each patient.

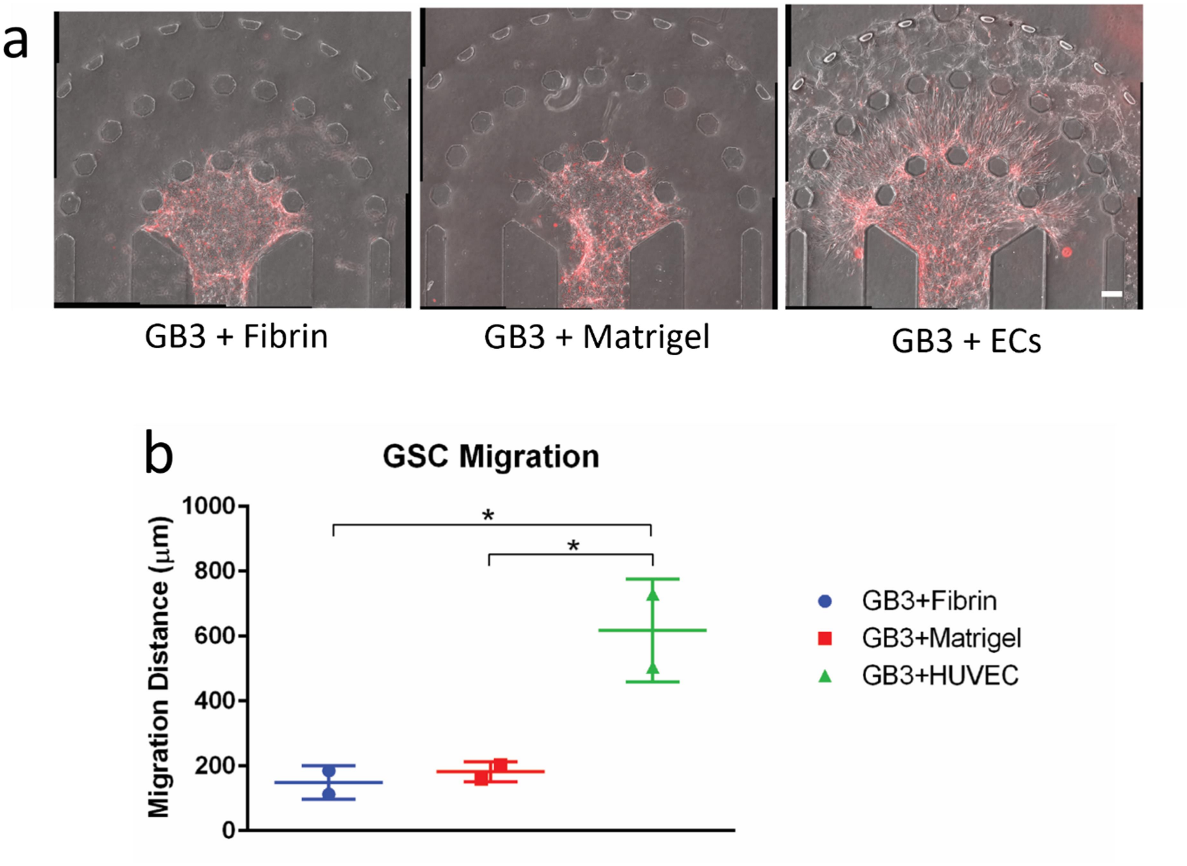

Supplementary Figure 7. Fibrin and Matrigel® matrix do not influence GB3-RFP cell migration at day 3. (A) Similar migratory tendencies of GB3-RFP cells were observed when fibrin was injected into the vascular region of the device, compared to the GB3-RFP mono-culture condition including Matrigel® in the vascular region. **(B)** In the presence of a vascular network, GB3-RFP cells achieve significant migration, compared to mono-culture conditions. Scale bar = 100 μm.

Supplementary Table 1: *Summary of experimental conditions in GBM tumor-on-chip platform*

	**Tumor layer**		**Stroma layer**		**Vascular layer**	
	Cell	*Matrix*	*Cell*	*Matrix*	*Cell*	*Matrix*
**Mono-culture**	GB3-RFP	Matrigel®	N/A	Matrigel®	N/A	Matrigel®
**Co-culture with Astrocytes**	GB3-RFP	Matrigel®	Astrocytes	Matrigel®	N/A	Matrigel®
**Co-culture with Vasculature**	GB3-RFP	Matrigel®	N/A	Matrigel®	Endothelial cells	Fibrin
**Tri-culture**	GB3-RFP	Matrigel®	Astrocytes	Matrigel®	Endothelial cells	Fibrin
